# Equid infective *Theileria* cluster in distinct *18S* rRNA gene clades comprising multiple taxa with unusually broad mammalian host ranges

**DOI:** 10.1186/s13071-020-04131-0

**Published:** 2020-05-19

**Authors:** Richard P. Bishop, Lowell S. Kappmeyer, Cynthia K. Onzere, David O. Odongo, Naftaly Githaka, Kelly P. Sears, Donald P. Knowles, Lindsay M. Fry

**Affiliations:** 1 Department of Veterinary Microbiology and Pathology, Washington State University, P.O. Box 647040, Pullman, Washington 99164 USA; 2 Animal Disease Research Unit, Agricultural Research Service, US Department of Agriculture, P.O. Box 646630, Pullman, WA 99164 USA; 3 Biosciences Eastern and Central Africa-International Livestock Research Institute (BecA-ILRI) Hub, P.O. Box 30709, Nairobi, 00100 Kenya; 4 School of Biological Sciences, University of Nairobi, P.O. Box 30197, Nairobi, 00100 Kenya

**Keywords:** Equine theileriosis, Clades, Phylotypes, *Theileria haneyi*, *Theileria equi*

## Abstract

Equine theileriosis, a tick-transmitted disease caused by the hemoprotozoan parasites *Theileria equi* and *Theileria haneyi*, affects equids throughout tropical and subtropical regions of the world. It is a significant regulatory concern in non-endemic countries, where testing for equine theileriosis is required prior to horse import to prevent parasite entry. Within endemic areas, infection causes significant morbidity and mortality, leading to economic losses. No vaccine for equine theileriosis is available, and current drug treatment protocols are inconsistent and associated with significant side effects. Recent work has revealed substantial genetic variability among equine theileriosis organisms, and analysis of ribosomal DNA from affected animals around the world indicates that the organisms can be grouped into five distinct clades. As these diverse parasites are capable of infecting a wide range of both tick and mammalian hosts, movement of different equine *Theileria* species between endemic countries, and eventually into non-endemic countries, is a significant concern. Furthermore, the substantial genetic variability of these organisms will likely render currently utilized importation diagnostic tests unable to detect all equine *Theileria* spp. To this end, more complete characterization of these diverse parasites is critical to the continued global control of equine theileriosis. This review discusses current knowledge of equine *Theileria* spp. in this context, and highlights new opportunities and challenges for workers in this field.
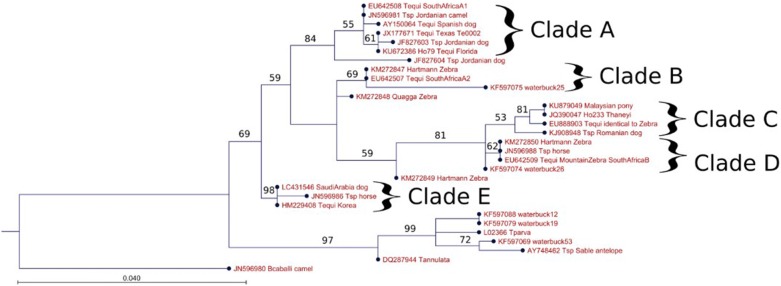

## Background

Equine theileriosis, caused by *Theileria equi* and *Theileria haneyi*, is common throughout tropical and subtropical regions of the world, and greatly constrains international movement of horses [1]. To prevent disease entry, testing of horses for *T. equi* is required prior to import into non-endemic countries. Furthermore, in equine theileriosis-endemic countries, in which over 90% of the world’s horses reside [2], economic losses due to morbidity and mortality are significant [3], and prevention of disease *via* vaccination is highly desirable, but not yet possible. Through improved surveillance and molecular technology, our understanding of the diversity of equine *Theileria* spp. parasites is rapidly expanding and is critical to the continued development of effective diagnostic and preventive strategies. Herein, we review current research regarding the genetic complexity of equine theileriosis, highlighting knowledge gaps and new research opportunities.

## The genus *Theileria*

The phylum Apicomplexa is a diverse and ancient one; some studies suggest it is approximately 800 million years old [4, 5]. The best-known members are mosquito-transmitted malaria parasites in the genus *Plasmodium*. Apicomplexans are defined by an apical complex of organelles, including rhoptries, micronemes and microspheres (also known as dense granules and spherical bodies). In many of the most economically and medically important genera, such as *Theileria*, *Toxoplasma*, *Plasmodium* and *Babesia*, the apical complex is necessary for invasion of mammalian host cells and subsequent intracellular establishment of the parasite [6–9].

Apicomplexan parasites in the genus *Theileria* are transmitted by ticks and can be divided into transforming and non-transforming types. The transforming *Theileria*, which infect and immortalize mammalian host leukocytes, resulting in a “cancer-like” phenotype, include the highly pathogenic *T. parva*, *T. annulata* and *T. lestoquardi.* Other host leukocyte-transforming species, such as *T. taurotragi*, a parasite of Eland, bushbuck and cattle, are less pathogenic. Transforming *Theileria* spp. undergo schizogony in concert with transformed host cell proliferation, which subsequently gives rise to an intraerythrocytic stage and a sexual cycle in the tick vector. In these parasitic infections, clinical disease is secondary to host cell transformation and the resultant inflammatory immune response [10, 11].

In contrast, non-transforming *Theileria* species, including the equine parasites *T. equi* and *T. haneyi*, and bovine parasites *T. orientalis* and *T. mutans*, have only a transient nucleated cell stage and persist as intra-erythrocytic piroplasms [12–15]. In these infections, clinical disease is secondary to red blood cell destruction and resultant anemia. Due to the prominent intraerythrocytic stage, transplacental transmission [16, 17], as well as iatrogenic transmission *via* contaminated needles and/or blood products [18–20], are common means of transmission in non-transforming *Theileria* species.

## Equine *Theileria* species

**Fig. 1:**
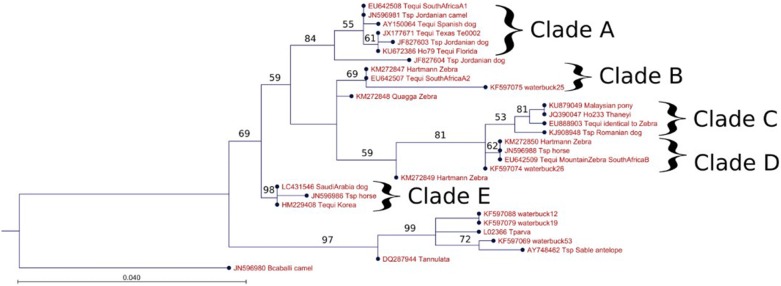
*18S* rDNA sequences placed in GenBank and identified as *Theileria equi* but detected within various host species were gathered and aligned in CLC Genomics Workbench v.10.1.1 (Qiagen), along with outgroup *18S* rDNA sequences from other piroplasmids as identified in the figure. All sequences were trimmed to identical length of 401 bp, and span the 4th hypervariable region of full-length *18S* rDNA. Aligned sequences were compared in a maximum likelihood phylogeny in the same program, with a Kimura 80 nucleotide substitution model, with neighbor-joining used in constructing initial trees to enable enhanced evolutionary inference. Five hundred replicates were performed in a bootstrap analysis with the values for nodes above a cut-off of 50 indicated on the tree for major branches. The tree provides a representative sample of ribosomal sequences falling into distinct clades across different infected host species and is not is intended to provide an exhaustive depiction of the relationships of all equid *Theileria* isolates

The *18S* ribosomal RNA gene sequences of equid-infective *Theileria* species separate into five clades (Fig. 1), which are discussed in detail later in this review. For the purposes of this review, parasites in rDNA clade A comprise *T. equi* (*sensu stricto*) which are the best characterized in terms of biology and economic importance, while those in clades B–E, (including *T. haneyi* in clade C) are designated *T. equi* (*sensu lato*).

Comparative genomics studies [12, 21] indicate that *T. equi* and *T. haneyi* have features intermediate between *Theileria* and *Babesia* and may represent a distinct taxon, closer to *Theileria* than *Babesia*. Similar to all other *Theileria* spp., *T. equi* undergoes schizogony in leukocytes prior to the development of intraerythrocytic stages [22], unlike *Babesia* spp., which infect only mammalian erythrocytes. An intraleukocytic life-cycle stage has not yet been demonstrated for *T. haneyi*, and research is underway to determine whether one exists.

Ultrastructural analysis of *Theileria* spp. that have so far been studied shows that the mammalian cell entry involves ‘zippering’ of closely juxtaposed parasite and host membranes, but does not require orientation dependent binding of the apical complex and release of the organelle contents, as in *Babesia* spp. and species of most other apicomplexan genera [6, 7]. Furthermore, in *Theileria*, the rhoptries and other apical organelles are involved in establishment within the host cell, but not in the entry process as in most Apicomplexa. The role of apical complex organelles in *T. equi* and *T. haneyi* host cell entry is currently unknown; however, unlike *T. parva* and *T. annulata*, *T. equi* sporozoites and merozoites possess morphological structures similar to micronemes [23] and may therefore utilize a different mechanism of host cell invasion. One important mechanism of protective immunity to transforming *Theileria*, including *T. parva*, depends both on the development of a CD8+ T-cell response to schizont infected host leukocytes and an antibody response to sporozoite antigens [24, 25]. While non-transforming *Theileria* also have a brief period of schizogony in host leukocytes [22], the existence and significance of a cell-mediated immune response to this parasite stage remains uncharacterized. Unlike the better-known transforming *Theileria* species, but similar to *Babesia*, transmission of *T. equi* in ticks occurs both transtadially and intrastadially [1].

In contrast to other *Theileria*, *T. equi-*type parasites infect multiple families of mammals, with reports of infection in dogs [26, 27], camels [28], tapirs [29], waterbuck, cattle, goats [30], and sheep [31] in addition to equids. Clinical disease has been observed in tapirs and dogs, but not in camels. Most other transforming and non-transforming *Theileria* species parasitize only a single mammalian family and frequently only one to three species within that family [32]. *Theileria equi* is also promiscuous in the range of ixodid tick vectors utilized, with at least 14 phylogenetically divergent tick species in four different genera implicated in natural and experimental transmission [2]. The ability to survive in many species of mammals and arthropod vectors may be a consequence of the larger complement of genes in *T. equi*, whose 11.7 megabase (Mb) genome contains 1985 predicted protein-encoding genes that are absent in *Babesia bovis* and host cell-transforming *Theileria* species [12]. *Theileria haneyi* has an approximately 10 Mb genome, intermediate in size between host cell-transforming *Theileria* and *T. equi.* This species has a minimum of 878 genes that are absent in *T. parva* and *T. annulata* [33].

Novel features of the *T. equi* genome include major expansions of the multicopy ATP binding cassette transporter protein family and the type II secretory protein family [21], which may contribute to the ability of the parasite to survive in multiple host and vector tissues. Many of the additional genes in *T. equi* and *T. haneyi* are located in paralogous multicopy gene families that exhibit no significant sequence identity to proteins in the public databases, although some contain conserved domains. Improved understanding of the molecular basis of promiscuous host and vector preferences therefore awaits a system for genetic manipulation and phenotyping of these parasites. Equid infective *Theileria* spp. possess FAINT (frequently-associated in *Theileria*) domains in multiple open reading frames (ORFS). FAINT domains, whose function is unknown, are present in the virtual proteomes of *T. parva*, *T annulata* and *T. orientalis*, but are absent in those of *Babesia* species [34, 35].

## The EMA gene family of *T. equi* and *T. haneyi*

An important feature common to *T. equi* and the newly discovered *T. haneyi* is a multicopy gene family encoding an immunodominant family of proteins believed to be involved in host erythrocyte invasion and/or exit. These are known as equine merozoite antigen (EMA) proteins, of which there are nine family members (EMA1-9) in both species that are thought to have evolved by gene duplication [21]. By contrast, the genomes of *T. parva*, *T. annulata* and *T. orientalis* each contain only single-copy genes that exhibit synteny with EMA5 in the genomes of the two equine *Theileria* species [33]. EMA5 is therefore likely orthologous to the single copy homologues in other *Theileria* species. However, whether EMA5 remains functionally equivalent, given the expansion of the EMA family in equid-infective *Theileria*, is unknown. Three copies of the family in *T. equi*, including EMA1, are absent from equivalent positions in the genome of *T. haneyi* according to syntenic analysis. However, the genome of *T. haneyi* contains three additional homologous genes with significant identity to the EMA family, but in different genomic locations. These copies are believed to have evolved more recently, and preserve the number of EMA genes [12]. Whether a complement of nine EMA genes is more widely conserved within the complex of *Theileria* species that are infective to equids and the possible functional divergence of the paralogous copies awaits further analysis. However, the ratio of non-synonymous to synonymous mutations in the EMA family indicates selection for conservation at certain residues, suggesting functional importance [33]. In *T. equi*, immune response research has focused almost entirely on the humoral immune response to EMA family antigens [1, 33, 36]. The situation is similar in *T. orientalis*, where almost all immune response data is centered on the immunodominant EMA orthologue, major piroplasm surface protein (MPSP) [37–39]. While infected animals develop robust humoral immune responses to these antigens, the responses are largely non-protective. Wise et al. [33] recently showed that the conservation of the EMA family present within the genomes of *T. equi* and *T. haneyi*, is probably not a response to immune pressure from the host. Thus, significant work is still required to obtain an understanding of the nature of protective immunity to non-transforming *Theileria* and its role in parasite evolution and genome diversification.

## Phylogeny of equine *Theileria* species

Parasites that have been classified under the umbrella term ‘*Theileria equi*’ infect zebra, domestic horses and donkeys [40, 41]. The *18S* rRNA gene sequences of equine *Theileria* fall into either four or five clades based on phylogenetic analysis of hypervariable region 4, depending on how a cluster is defined [42–45]. Using a maximum likelihood algorithm, following initial implementation of a neighbor-joining approach, we identify five clades in our analysis with clades C and D being relatively similar (Fig. 1, clades A–E). *Theileria* sequences were selected for inclusion in this analysis from the NCBI nucleotide database by searching for ‘*Theileria equi 18S*’ and then choosing sequence derived from hypervariable region 4 based on diversity of the mammalian host species listed as the source of the sample. Redundant sequences from the same host and from the same locality were reduced to one representative. Selection was done without regard for whether the sequence was from a published journal article and because accession numbers are provided in the tree, no further references were added to the citations. The ribosomal RNA gene clade containing *T. parva* and *T. annulata* is also shown in Fig. 1 in order to provide a scale illustrating the distinctness of the equine-infective *Theileria* clusters. Co-infection of horses with equine *Theileria* spp. from different clades was recently documented experimentally [46] and, importantly, in horses and donkeys from the field in South Africa [42] and the Gambia [47], as well as wild zebra from South Africa [42]. Interestingly, *Theileria* spp. diversity was significantly greater in zebra, with clades A, B, C and D all detected in South African mountain zebra (*Equus zebra*) and several in Burchell’s zebra (*Equus quagga*), suggesting that zebra could be an ancestral mammalian host of parasites causing equine theileriosis [42].

Clade A contains *Theileria equi* (*sensu stricto*), which causes infections of variable severity, but is capable of inducing severe anemia and resultant mortality in domestic equids. In most instances, however, the incalculable cost of subclinical morbidity and reduced performance of animals used for transport, traction and racing far surpasses the impact of disease mortality [1]. The parasite, which is endemic throughout most of Africa, mainland Europe, the Middle East and Latin America [2], has recently been detected in the UK, previously thought to be disease-free [47]. Diagnosis of *T. equi* (*sensu stricto*), which is implemented using a competitive ELISA based on the antigen EMA1, is important for regulation of the global movement of horses [1]. Thus, genetic variation from *T. equi* (*sensu stricto*), especially at the EMA1 locus, can significantly hinder detection of such organisms with the currently available diagnostic assays. For instance, due to its complete lack of the EMA1 gene, *T. haneyi* is not detected by the *T. equi* competitive ELISA test [8]. Other divergent equine-infective *Theileria* species could cause similar problems.

Equid apicomplexan *18S* rRNA clades C and D, which are genetically relatively similar to one another based on clustering of nodes, as compared to clade A, contain the recently described, novel *Theileria* species, *Theileria haneyi*, which was discovered in horses in the USA [12] and subsequently identified in horses from South Africa [42]. The presumptive timescale of speciation of the two fully sequenced equine *Theileria* species, *T. equi* and *T. haneyi*, is estimated at 33 million years [12]. This speciation event long precedes emergence of the only surviving equid genus, *Equus*, 4–4.5 million years ago [48]. Clades C and D also contain parasites present in common zebra (*Equus quagga*) and both subspecies of mountain zebra (*Equus zebra)*, as well as dogs. Surprisingly, waterbuck (*Kobus ellipsiprymnus defassa*) [49], which is in the family Bovidae, and is evolutionarily distinct from equids, was also infected with a clade C parasite. A highly similar ribosomal DNA sequence was also identified in parasites from a Malaysian pony (Fig. 1), demonstrating that *18S* rRNA phylotypes related to *T. haneyi* are present on three continents. Remarkably, the average predicted protein sequence divergence between the genomes of *T. equi* (Florida isolate) and *T. haneyi* (23%), is greater than that of the geographically separated, host cell-transforming *T. parva* and *T. annulata* (18%). *Theileria parva* and *T. annulata* also differ in wildlife host, mammalian cell tropism and tick vector. While the exact percentage difference between protein sequences in the two equid-infective *Theileria* may alter as annotation is improved through new data resources, such as transcriptomes, these differences suggest functional divergence in at least some of these proteins.

Equid apicomplexan rRNA clade B contains parasites identified in mountain zebra, common zebra, South African domestic horses, and East African waterbuck, the latter originating from the same geographical region as the isolate in clade C (Fig. 1). The complete genome sequence is not yet available for any of the ribosomal phylotypes that cluster in clade B, but it is reasonable to assume based on the extent of genome difference between *T. equi* and *T. haneyi*, that these are likely to be distinct at the whole genome level from the parasites clustered in other clades. The equid *Theileria* rRNA clade E so far only contains parasites from domestic horses (*Equus caballus)* in two widely separated countries, Korea and Jordan, together with a parasite detected in a dog from Saudi Arabia (Fig. 1). Similar to clade B, there is as yet no complete genome sequence originating from parasites within this ribosomal phylotype. However, the level of difference in the *18S* rDNA sequence again suggests that there could be significant evolutionary divergence at the genome level. No wildlife mammalian reservoir has been identified for this group yet. Interestingly, parasites from three of the five *18S* clades, namely A, C and E, have been identified in samples from dogs in Europe and the Middle East (Fig. 1), although nothing is yet known about the infection dynamics or biology of *T. equi* in canids. As was recently discussed [50], rRNA sequences are important for defining potential novel taxa and identifying new avenues of investigation of equid *Theileria* biology and infection dynamics. However, in future they need to be supported by more conventional investigation of transmission and biology in the mammalian host and tick vectors to ascertain the significance of rRNA phylotype diversity in equids and additional ‘hosts’. Additionally, *18S* rRNA sequences, despite their pedigree as phylogenetic markers, represent a relatively small part of the overall parasite genetic complement. They should therefore be supported by complete genome sequences, which are more definitive and informative.

Parasites from members of several clades that have been classified as *T. equi* (*sensu lato*) frequently induce long-term asymptomatic infections in zebra. One hundred percent of Grevy’s zebra (*Equus grevyi*) sampled in Kenya were found to be positive for *T. equi-*type parasites using PCR with *18S* rRNA primers. Similarly, 100% of Cape mountain zebra (*Equus zebra zebra)* in South Africa were positive using a multiplex real-time PCR assay [51, 52]. Common zebra (*Equus quagga*) are also infected in East Africa according to *18S* rRNA PCR data (DOO and RPB, unpublished data), and a similar high prevalence of infection was observed in asymptomatic zebra in South Africa, with two distinct clades represented within the V4 hypervariable region of the *18S* gene [44].

*Theileria haneyi* does not cause severe disease following experimental infection of normal horses [46], which may explain why it has remained ‘cryptic’ for so long, despite a global distribution. The same may also be true of the parasites in equid-infective ribosomal clades B, D and E, whose full distribution has yet to be systematically investigated. Recent studies of non-pathogenic *T. mutans* and *T. velifera* infections of cattle have highlighted the potential importance of co-infections in modulating disease outcomes for *T. parva* [53]. The same may be true for equid-infective apicomplexan parasites, as recent studies have revealed that *T. equi* and *T. haneyi* can co-infect horses [46]. Studies to determine whether clinical response to infection and efficacy of treatment differs in hosts that are co-infected with *T. equi* and *T. haneyi*, as compared to those infected with only a single species, are currently underway. These issues are important, given that the recent surveys of the prevalence of different *T. equi* rRNA gene clades in the field in South Africa and the Gambia revealed that co-infection is frequent [42, 47].

Given the complexities in the phylogeny and infection dynamics of equid *Theileria* spp., an improved understanding of the prevalence and diversity of all clades within this group of parasites is critical to improved global control of equine theileriosis.

## Conclusions

Equid *Theileria* are promiscuous, with infections so far also documented in camelidae, tapiridae, bovidae and canidae. Further monitoring of prevalence, with a focus on co-infections, accompanied by generation of additional genome sequences from equid-infective parasites with distinct ribosomal phylotypes is therefore a priority for future research. Currently, there are insufficient whole genomes to allow meaningful application of comparative genomics to this group of parasite species. As global warming expands the habitat of competent tick vectors, spread of tick-borne disease is inevitable. Furthermore, with the growing development of resistance to acaricides and the growing public concern regarding chemically mediated control [54–57], a more complete understanding of the comparative genomics of equine *Theileria* spp. is crucial to improve global control of equine theileriosis with diagnostics and vaccination. In addition to the issue of host and vector range, a particular area of interest in *Theileria* is the relationship between infection and pathology. For example, among the equid-infective parasites, *T. equi* induces more severe hemolytic symptoms in horses than *T. haneyi*, but the underlying virulence factors encoded by these parasites are as yet unknown. Unlike other *Theileria* species, the erythrocyte infective stage of *T. equi* (*sensu stricto*) can be cultured *in vitro*, although this is not yet possible for *T. haneyi*. Thus, *T. equi* could serve as a model for development of parasite transfection systems that will enable functional analysis and identification of virulence factors in this fascinating group of pathogens.

## Data Availability

Not applicable.

## Abbreviations

RNA: Ribonucleic acid
rRNA: Ribosomal RNA
Mb: Megabase
ATP: Adenosine triphosphate
FAINT: Frequently associated in *Theileria*
ORF: Open reading frame
EMA protein: Equine merozoite associated protein
MPSP: Major piroplasm surface protein
PCR: Polymerase chain reaction

## References

[1] Wise LN, Kappmeyer LS, Mealey RH, Knowles DP (2013). Review of equine piroplasmosis. J Vet Intern Med..

[2] Scoles GA, Ueti MW (2015). Vector ecology of equine piroplasmosis. Annu Rev Entomol..

[3] Rothschild CM (2013). Equine piroplasmosis. J Equine Vet Sci..

[4] Levine N, Phylum II, Lee JJ, Hutner SH, Bovie EC (1985). Apicomplexa Levine, 1970. An illustrated guide to the protozoa.

[5] Escalante AA, Ayala FJ (1995). Evolutionary origin of *Plasmodium* and other Apicomplexa based on rRNA genes. Proc Natl Acad Sci USA.

[6] Sam-Yellowe TY (1996). Rhoptry organelles of the Apicomplexa: their role in host cell invasion and intracellular survival. Parasitol Today..

[7] Shaw MK (2003). Cell invasion by *Theileria* sporozoites. Trends Parasitol..

[8] Kemp LE, Yamamoto M, Soldati-Favre D (2013). Subversion of host cellular functions by the apicomplexan parasites. FEMS Microbiol Rev..

[9] Carruthers VB, Sibley L (1997). Sequential protein secretion from three distinct organelles of *Toxoplasma gondii* accompanies invasion of human fibroblasts. Eur J Cell Biol..

[10] Fry LM, Schneider DA, Frevert CW, Nelson DD, Morrison WI, Knowles DP (2016). East coast fever caused by *Theileria parva* is characterized by macrophage activation associated with vasculitis and respiratory failure. PLoS ONE..

[11] Tretina K, Gotia HT, Mann DJ, Silva JC (2015). *Theileria*-transformed bovine leukocytes have cancer hallmarks. Trends Parasitol..

[12] Knowles DP, Kappmeyer LS, Haney D, Herndon DR, Fry LM, Munro JB (2018). Discovery of a novel species, *Theileria haneyi* n. sp., infective to equids, highlights exceptional genomic diversity within the genus *Theileria*: implications for apicomplexan parasite surveillance. Int J Parasitol..

[13] Sivakumar T, Hayashida K, Sugimoto C, Yokoyama N (2014). Evolution and genetic diversity of *Theileria*. Infect Genet Evol..

[14] Schein E, Rehbein G, Voigt WP, Zweygarth E (1981). *Babesia equi* (Laveran, 1901) 1. Development in horses and in lymphocyte culture. Tropenmed Parasitol..

[15] Moltmann UG, Mehlhorn H, Schein E, Rehbein G, Voigt WP, Zweygarth E (1983). Fine structure of *Babesia equi* (Laveran, 1901) within lymphocytes and erythrocytes of horses: an *in vivo* and *in vitro* study. J Parasitol..

[16] Mekata H, Minamino T, Mikurino Y, Yamamoto M, Yoshida A, Nonaka N (2018). Evaluation of the natural vertical transmission of *Theileria orientalis*. Vet Parasitol..

[17] Sant C, d’Abadie R, Pargass I, Basu AK, Asgarali Z, Charles RA (2016). Prospective study investigating transplacental transmission of equine piroplasmosis in thoroughbred foals in Trinidad. Vet Parasitol..

[18] Hammer JF, Jenkins C, Bogema D, Emery D (2016). Mechanical transfer of *Theileria orientalis*: possible roles of biting arthropods, colostrum and husbandry practices in disease transmission. Parasit Vectors..

[19] Yona S, Kim KW, Wolf Y, Mildner A, Varol D, Breker M (2013). Fate mapping reveals origins and dynamics of monocytes and tissue macrophages under homeostasis. Immunity..

[20] Gerstenberg C, Allen W, Phipps L. Mechanical transmission of *Babesia equi* infection in a British herd of horses. In: Proceedings of the 8th International Conference of Equine Infectious Diseases, 23–26 March, Dubai, UAE; 1998.

[21] Kappmeyer LS, Thiagarajan M, Herndon DR, Ramsay JD, Caler E, Djikeng A (2012). Comparative genomic analysis and phylogenetic position of *Theileria equi*. BMC Genomics..

[22] Ramsay JD, Ueti MW, Johnson WC, Scoles GA, Knowles DP, Mealey RH (2013). Lymphocytes and macrophages are infected by *Theileria equi*, but T cells and B cells are not required to establish infection *in vivo*. PLoS ONE..

[23] Mehlhorn H, Schein E (1998). Redescription of *Babesia equi* Laveran, 1901 as *Theileria equi* Mehlhorn, Schein 1998. Parasitol Res..

[24] McKeever DJ, Taracha EL, Innes EL, MacHugh ND, Awino E, Goddeeris BM (1994). Adoptive transfer of immunity to *Theileria parva* in the CD8+ fraction of responding efferent lymph. Proc Natl Acad Sci USA.

[25] Musoke AJ, Nantulya VM, Buscher G, Masake RA, Otim B (1982). Bovine immune response to *Theileria parva*: neutralizing antibodies to sporozoites. Immunology..

[26] Beck R, Vojta L, Mrljak V, Marinculic A, Beck A, Zivicnjak T (2009). Diversity of *Babesia* and *Theileria* species in symptomatic and asymptomatic dogs in Croatia. Int J Parasitol..

[27] Rosa CT, Pazzi P, Nagel S, McClure V, Christie J, Troskie M (2014). Theileriosis in six dogs in South Africa and its potential clinical significance. J S Afr Vet Assoc..

[28] Qablan MA, Sloboda M, Jirků M, Oborník M, Dwairi S, Amr ZS (2012). Quest for the piroplasms in camels: identification of *Theileria equi* and *Babesia caballi* in Jordanian dromedaries by PCR. Vet Parasitol..

[29] Da Silveira AW, De Oliveira GG, Menezes Santos L, da Silva Azuaga LB, Macedo Coutinho CR, Echeverria JT (2017). Natural infection of the South American tapir (*Tapirus terrestris*) by *Theileria equi*. J Wildl Dis..

[30] Zhang J, Kelly P, Li J, Xu C, Wang C (2015). Molecular detection of *Theileria* spp. in livestock on five Caribbean islands. Biomed Res Int..

[31] Azmi K, Al-Jawabreh A, Abdeen Z (2019). Molecular detection of *Theileria ovis* and *Theleiria equi* in livestock from Palestine. Sci Rep..

[32] Bishop R, Musoke A, Morzaria S, Gardner M, Nene V (2004). *Theileria*: intracellular protozoan parasites of wild and domestic ruminants transmitted by ixodid ticks. Parasitology..

[33] Wise LN, Kappmeyer LS, Knowles DP, White SN (2019). Evolution and diversity of the EMA families of the divergent equid parasites, *Theileria equi* and *T. haneyi*. Infect Genet Evol..

[34] Pain A, Renauld H, Berriman M, Murphy L, Yeats CA, Weir W (2005). Genome of the host-cell transforming parasite *Theileria annulata* compared with *T. parva*. Science..

[35] El-Sayed SAE, Rizk MA, Terkawi MA, Mousa A, Elsayed G, Fouda M (2015). Cocktail of *Theileria equi* antigens for detecting infection in equines. Asian Pac J Trop Biomed..

[36] Silva MG, Graça T, Suarez CE, Knowles DP (2013). Repertoire of *Theileria equi* immunodominant antigens bound by equine antibody. Mol Biochem Parasitol..

[37] Zhuang W, Sugimoto C, Matsuba T, Niinuma S, Murata M, Onuma M (1994). Analyses of antigenic and genetic diversities of *Theileria sergenti* piroplasm surface proteins. J Vet Med Sci..

[38] Onuma M, Kakuda T, Sugimoto C (1998). *Theileria* parasite infection in East Asia and control of the disease. Comp Immunol Microbiol Infect Dis..

[39] Jenkins C, Bogema DR (2016). Factors associated with seroconversion to the major piroplasm surface protein of the bovine haemoparasite *Theileria orientalis*. Parasit Vectors..

[40] Schein E, Ristic M (1988). Equine babesiosis. Babesiosis of domestic animals and man.

[41] Friedhoff KT, Tenter AM, Muller I (1990). Haemoparasites of equines: impact on international trade of horses. Rev Sci Tech..

[42] Bhoora RV, Collins NE, Schnittger L, Troskie C, Marumo R, Labuschagne K (2019). Molecular genotyping and epidemiology of equine piroplasmids in South Africa. Ticks Tick Borne Dis..

[43] Peckle M, Pires MS, Silva CBD, Costa RLD, Vitari GLV, Senra MVX (2018). Molecular characterization of *Theileria equi* in horses from the state of Rio de Janeiro, Brazil. Ticks Tick Borne Dis..

[44] Bhoora R, Buss P, Guthrie AJ, Penzhorn BL, Collins NE (2010). Genetic diversity of piroplasms in plains zebra (*Equus quagga burchellii*) and Cape mountain zebra (*Equus zebra zebra*) in South Africa. Vet Parasitol..

[45] Salim B, Bakheit MA, Kamau J, Nakamura I, Sugimoto C (2010). Nucleotide sequence heterogeneity in the small subunit ribosomal RNA gene within *Theileria equi* from horses in Sudan. Parasitol Res..

[46] Sears KP, Kappmeyer LS, Wise LN, Silva M, Ueti MW, White S (2019). Infection dynamics of *Theileria equi* and *Theileria haneyi*, a newly discovered apicomplexan of the horse. Vet Parasitol..

[47] Coultous RM, Phipps P, Dalley C, Lewis J, Hammond TA, Shiels BR (2019). Equine piroplasmosis status in the UK: an assessment of laboratory diagnostic submissions and techniques. Vet Rec..

[48] Orlando L, Ginolhac A, Zhang G, Froese D, Albrechtsen A, Stiller M (2013). Recalibrating *Equus* evolution using the genome sequence of an early Middle Pleistocene horse. Nature..

[49] Githaka N, Konnai S, Bishop R, Odongo D, Lekolool I, Kariuki E (2014). Identification and sequence characterization of novel *Theileria* genotypes from the waterbuck (*Kobus defassa*) in a *Theileria parva*-endemic area in Kenya. Vet Parasitol..

[50] Uilenberg G, Gray J, Kahl O (2018). Research on Piroplasmorida and other tick-borne agents: are we going the right way?. Ticks Tick Borne Dis..

[51] Smith RM, Bhoora RV, Kotzé A, Grobler JP, Lee Dalton D (2019). Translocation a potential corridor for equine piroplasms in Cape mountain zebra (*Equus zebra zebra*). Int J Parasitol Parasites Wildl..

[52] Hawkins E, Kock R, McKeever D, Gakuya F, Musyoki C, Chege SM (2015). Prevalence of *Theileria equi* and *Babesia caballi* as well as the identification of associated ticks in sympatric Grevy’s zebras (*Equus grevyi*) and donkeys (*Equus africanus asinus*) in northern Kenya. J Wildl Dis..

[53] Woolhouse ME, Thumbi SM, Jennings A, Chase-Topping M, Callaby R, Kiara H (2015). Co-infections determine patterns of mortality in a population exposed to parasite infection. Sci Adv..

[54] Stone NE, Olafson PU, Davey RB, Buckmeier G, Bodine D, Sidak-Loftis LC (2014). Multiple mutations in the para-sodium channel gene are associated with pyrethroid resistance in *Rhipicephalus microplus* from the United States and Mexico. Parasit Vectors.

[55] Eiden AL, Kaufman PE, Oi FM, Allan SA, Miller RJ (2015). Detection of permethrin resistance and fipronil tolerance in *Rhipicephalus sanguineus* (Acari: Ixodidae) in the United States. J Med Entomol..

[56] Aenishaenslin C, Michel P, Ravel A, Gern L, Waaub JP, Milord F (2016). Acceptability of tick control interventions to prevent Lyme disease in Switzerland and Canada: a mixed-method study. BMC Public Health..

[57] Adalja A, Sell ST, McGinty M, Boddie C (2016). Genetically modified mosquito use to reduce mosquito-transmitted disease in the US: a community opinion survey. PLoS Curr..

